# Exposure to tobacco smoke and validation of smoking status during pregnancy in the MIREC study

**DOI:** 10.1038/s41370-017-0011-z

**Published:** 2018-01-03

**Authors:** Tye E. Arbuckle, Chun Lei Liang, Mandy Fisher, Nicolas J. Caron, William D. Fraser, and the MIREC Study Group

**Affiliations:** 1 Population Studies Division, Environmental Health Science and Research Bureau, Healthy Environments and Consumer Safety Branch, Health Canada, Ottawa, ON Canada; 2 Le Centre de toxicologie du Québec, Institut national de santé publique du Québec, Quebec, QC Canada; 3 Centre de recherche du Centre hospitalier universitaire de Sherbrooke, Sherbrooke, QC Canada; 4 Sainte Justine University Hospital Research Center, University of Montreal, Montreal, QC Canada

**Keywords:** Biomonitoring, Plasma, Meconium, Cotinine, Smoking, Pregnancy, Multiunit dwelling

## Abstract

Given that prenatal exposure to tobacco smoke can lead to increased risks of adverse health effects, having valid measures of exposure is important. In a Canadian cohort (*n* = 2000), maternal and infant biospecimens were analysed for cotinine. Sensitivity and specificity of self-reported active smoking status were estimated. Regression modelling was used to identify potential predictors of maternal and infant plasma cotinine in non-smoking women. During the first trimester, 60.6% of the women reported never smoking, 27.3% were former smokers, 6.1% had quit when they found out they were pregnant, 5.8% were smokers and 42% of the non-smokers reported exposure to secondhand smoke (SHS). Low detection of tobacco biomarkers in meconium limited its ability to identify exposure to SHS. The sensitivity and specificity for self-reported smoking during the 1st trimester were 85.37 and 99.45%, respectively. The lowest sensitivity was found in participants with the highest level of education and income, oldest women and those born outside Canada. Non-smoking women living in an apartment had 1.7 times higher odds of detectable plasma cotinine than those living in a single home after adjusting for other variables. Our results suggest that while self-reports are fairly accurate, they may be less so in populations with higher socio-economic status. This investigation underscores the need to consider the participant socio-economic characteristics and dwelling type when using questionnaires to estimate active and passive tobacco exposure.

## Introduction

A recent review has concluded that both exposures to active and secondhand tobacco smoke (SHS) during pregnancy can lead to adverse effects on the developing fetus affecting the endocrine, reproductive, respiratory, cardiovascular and neurological systems [1].

Underreporting of smoking status may result in seriously biased risk assessments. Given the costs of collecting and analysing multiple maternal biospecimens for cotinine, large epidemiologic studies routinely rely on maternal self-reports of exposure to tobacco smoke. As the validity of self-reports among pregnant women may vary by study population (for example, 25% underestimation of true smokers in a Scottish study [2], 6% in a Swedish study [3] and 2% in the Norwegian MoBa cohort [4]), it is important to compare self-reported smoking status with cotinine measurements for the population under study.

In pregnancy, the metabolic clearance of nicotine increases about 60%, whereas cotinine increases by 140%, substantially reducing cotinine’s half-life [5]. Although maternal serum, plasma or urinary cotinine have traditionally been the metric of choice to estimate prenatal exposure to tobacco smoke, biomarkers in meconium have also been measured [6, 7]. It is generally thought that meconium begins to form around the 13th week of gestation and thus can represent cumulative exposure over the final two trimesters [8].

There currently are no large biomonitoring datasets that measure exposure to tobacco in Canadian pregnant women and newborn infants. The present study was designed to measure exposure to tobacco smoke using both maternal self-reports and biomarkers and to assess the validity of self-reported smoking in this large Canadian prospective cohort of pregnant women. In addition, there was interest in identifying maternal characteristics associated with elevated plasma cotinine concentrations in non-smokers, especially from multiunit housing. Meconium was also evaluated as a matrix for measuring fetal exposure in a population with low prevalence of smoking.

## Subjects and methods

### Study population

Data and biological samples from the mothers and newborns participating in the Maternal-Infant Research on Environmental Chemicals (MIREC) Study were collected to measure exposure to tobacco smoke. MIREC is a national-level pregnancy cohort study that recruited 2001 women during the 1st trimester of pregnancy from 10 cities across Canada from 2008–2011. Further details on the cohort have been described previously [9]. The protocol and survey instruments were reviewed by the Health Canada Research Ethics Board and ethic committees at each participating recruitment site. All participants signed informed consent forms prior to enrolment in the study.

### Tobacco smoke exposure

#### Self-reported exposure

Questionnaires were administered to women during the 1st (T1) and 3rd (T3) trimester clinic visits by trained interviewers. To assess active tobacco smoke exposure, women were asked if they had ever smoked at least 100 cigarettes over their lifetime, whether they currently smoke daily or occasionally, how many cigarettes they usually smoke, and whether they had stopped smoking during the pregnancy or before they became pregnant. Exposure to secondhand smoke (SHS) was measured by the following questions: “Including both household members and regular visitors, does anyone smoke inside your home, every day or almost every day? (include cigarettes, cigars and pipes)”; “In the past month, were you exposed to SHS, every day or almost every day, in a car or other private vehicle?”; “During your pregnancy, has anyone in your workplace smoked in your presence? (including breaks, lunch)”; “During your pregnancy, were you exposed to SHS in public places? (such as bars, arenas, restaurants or bingo halls)”.

#### Biomarkers of exposure

##### Plasma

Plasma samples were collected at T1 and T3 and venous umbilical cord plasma at delivery. Samples were aliquotted and stored at −20 °C until transported to the Institut national de santé publique du Québec (INSPQ) laboratory for analysis which has ISO/IEC 17025 and CAN-P-43 accreditation.

Two methods with different sensitivities were used to measure plasma cotinine. Initially, the standard method aimed at determining active smoking status was used with a LOD of 0.4 ng/ml and a limit of linearity extending up to 500 ng/ml. When it became apparent that there was a high rate of non-detects among the samples, a more sensitive method (LOD < 0.005 ng/ml and a limit of linearity up to 10 ng/ml) was developed that could be used on samples from women who reported being non-smokers. Samples were analysed with the non-smokers method if our records indicated that they were non-smokers; otherwise the standard method was used to avoid carry-over issues and excessive repeat testing. If the woman reported being a non-smoker, samples previously analysed with the standard method, were re-analysed with the more sensitive method. However, in the early stages of MIREC, some biospecimens were destroyed after the analyses were completed as we had not anticipated the need to store leftover biospecimens. Therefore, about 305 1st trimester, 70 3rd trimester, and 40 cord bloods from self-reported non-smokers were not re-analysed.

For the standard method, free cotinine was extracted from plasma using automated mixed-mode solid-phase extraction in a 96-well format on a Janus robotic station (PerkinElmer; Waltham, Massachusetts, USA) (INSPQ method C-551). Extraction was performed using Oasis MCX 96-well plates (Waters; Milford, Massachusetts, USA) containing 30 mg mass of resin. The sorbent chemistry used in this extraction was designed for mixed-mode cation-exchange of basic drugs in acidified samples. In short, each 500 µl plasma sample was acidified with an equal volume of phosphoric acid (5%) after which the cotinine-d3 internal standard was added. The sample was then loaded on the pre-equilibrated solid-phase sorbent before a 2% formic acid aqueous wash, followed by a methanol wash. Samples were eluted using a 5% NH_4_OH/methanol solution before they were evaporated to dryness. The dried extracts were taken up in 200 µl of a solvent solution compatible with the mobile phase at the initial stage of the chromatography. They were then analyzed by LC–MS/MS with an Ultra Performance Liquid Chromatography (UPLC) Acquity (Waters) coupled with a tandem mass spectrometer Quattro Premier XE (Waters) in MRM mode with an electrospray ion source in positive mode. The analytical column used was an Acquity UPLC BEH C18, 2.1 × 50 mm, 1.7 µm (Waters). The validated limit of detection (LOD) for free cotinine was below 0.4 ng/mL for the duration of the study.

The free cotinine quantitation method for non-smokers (INSPQ method C-556) was adapted from the standard method with minor modifications, mainly, the use of a more sensitive mass spectrometer. In short, a 200 µl plasma sample was submitted to the same extraction procedure as described above, and at the end of the procedure taken up in 500 µl of mobile phase. Samples were then injected by LC–MS/MS with an UPLC Acquity (Waters) coupled with a Xevo TQ-S tandem mass spectrometer (Waters) in MRM mode with an electrospray ion source in positive mode. The analytical column used was an Acquity UPLC BEH C18, 2.1 × 50 mm, 1.7 µm (Waters). The LOD for free cotinine was below 0.005 ng/mL for the duration of the study.

The following calibration standards: (−)-Cotinine and (±)-Cotinine-d3 were acquired from Cerilliant (Round Rock, Tx, USA). The following MRM transition (*m/z*) were monitored for quantitation/qualifier ions: 177.1-80.1/177.1-98.0 for cotinine and 180.1-80.1 quantitation ion for the cotinine-d3 internal standard. Results from analysis of in-house reference materials are provided in the Supplemental Information.

##### Meconium

Meconium was collected within the first few days after birth and analysed for nicotine, cotinine and 3-hydroxycotinine. Meconium samples were analyzed using a method adapted from the standard smoker’s method for serum cotinine described above, with minor modifications. For meconium (INSPQ method E-570), samples were extracted using automated mixed-mode Oasis MCX 96-well plates (Waters) on a Janus robotic station (PerkinElmer) and analyzed with a Quattro Premier XE LC–MS/MS (Waters) in MRM mode with an electrospray ion source in positive mode. First, approximately 500 mg of each sample was weighed before homogenisation and suspension in methanol, followed by internal standards addition, acidification and vortexing. Each methanol suspended sample was acidified with an equal volume of phosphoric acid (4%) after internal standards were added. The analytical column used was an Acquity UPLC HSS T3, 2.1 × 50 mm, 1.8 µm (Waters). The following calibration standards: (−)-Cotinine, S(−)-Nicotine, (±)-Cotinine-d3 and (±)-Nicotine-d4 were acquired from Cerilliant, while trans-3′-Hydroxycotinine and trans-3′-Hydroxycotinine-d3 were acquired from Toronto Research Chemicals (Toronto, Ontario, Canada). All data was acquired in MRM mode with an electrospray ion source in positive mode. The following MRM transition (*m/z*) were monitored for quantitation/qualifier ions: 163.2-130.0/163.2-117.0 for nicotine, 177.1-80.1/177.1-98.0 for cotinine and m/z 193.1-80.0/193.1-134.0 for hydroxycotinine. The following MRM transition ions (*m/z*) were monitored for the internal standards: 167.3-134.1 for Nicotine-d4, 180.1-80.1 for cotinine-d3 and *m/z* 196.3-89.0 for Hydroxycotinine-d3.

Results were converted to ng/g of meconium. The method’s LODs for nicotine, cotinine and 3-hydroxycotinine in meconium were 0.13, 0.076, and 0.093 ng/g, respectively. The detection limits of the method were determined for a sample having a weight of 500 mg (weight required for the analysis). For samples with a weight of 500 mg and above, detection limits are those specified in the method. In cases where the sample weight was less than 500 mg, the analysis was performed and the detection limits were recalculated on the basis of weight, resulting in several LODs for a few of the meconium results.

##### Material testing

No cotinine was detected in the diaper liners, cord blood collection bags (PALL®), pipette tips, Sarstedt® or Falcon® tubes.

### Covariates

Maternal age, education, household income, parity, pre-pregnancy body mass index (BMI), dwelling type and woman’s country of birth were collected from the T1 questionnaire. Infant gender was abstracted from the medical charts.

### Statistical analysis

#### Descriptive statistics and hypothesis testing

As substitution methods such as ½ LOD are ad-hoc procedures that can lead to increased bias and an underestimation of the error variance, which results in low power for statistical hypothesis testing [10, 11], survival analysis techniques for left-censored data were applied to our biomonitoring data [11]. Descriptive statistics, including percentage of observations below the LOD, minimum, median, 95th percentile, maximum, geometric mean (GM), and associated 95% confidence interval were calculated. The GM from a lognormal random variable with censoring was calculated using the maximum likelihood (ML) estimation method and the empirical median from the Kaplan–Meier (KM) approach. The Greenwood estimate of variance was used for determination of KM confidence intervals. The censoring methods were used only for chemicals with at least 50% of the data above the LOD. Spearman correlations among various matrices for cotinine were calculated since the cotinine levels were not normally distributed.

Hypothesis testing for the covariates associated with nicotine metabolites was performed using the likelihood ratio test for parametric ML estimation. The Bonferroni multiple comparison technique was used when overall tests were significant for the parametric ML estimation method. The *p*-value obtained from the likelihood ratio test of the ML estimation method for each comparison was compared to an adjusted significance level of 0.05/g (g: number of comparisons) in order to achieve an overall significance level of *α* = 0.05.

#### Sensitivity and specificity of self-reports of active tobacco exposure

Active smokers (daily or occasionally) were separated from non-active (never, former or quit during pregnancy) smokers by estimating the lowest point between two distinct distributions of log plasma cotinine. A nonparametric bootstrap method was performed, whereby 10,000 alternative data sets were randomly resampled from the study population (Supplemental Figures). For each data set, the lowest log plasma cotinine point between two peaks was located using kernel density estimation. The mean and percentiles of the 10,000 point estimates were calculated and exponentiated back to original scale. The exponentiated mean was used as the plasma cotinine cut-off between active smokers and non-active smokers. The 95% CI of the plasma cotinine cut-off was constructed by the exponentiated 2.5th percentile and 97.5th percentile of the 10,000 estimates [4].

Sensitivity and specificity of self-reported active smoking status were estimated using the cotinine cut-off as determined by the bootstrap method. The estimated plasma cotinine cut-off was considered to be the “gold standard”, and self-reported active smoking status was considered the “test” in sensitivity and specificity calculations. Values less than the LOD were assigned a constant value LOD/2.

#### Predictors of plasma cotinine in non-smokers

Self-reported never and former smokers were considered as non-smokers for this analysis. As some women may have incorrectly reported that they were non-smokers (had an elevated plasma cotinine concentration), the cut-off point derived by the method described above (See section “Sensitivity and specificity of self-reports of active tobacco exposure”) was used to remove likely smokers from further analysis. Self-reported non-smokers were also excluded if the less sensitive cotinine analysis method was used.

Initially, univariate logistic regression models were used to assess the association between cotinine in maternal plasma and variables of interest. The response variable, T1 or T3 cotinine, was categorized as follows: (1) if cotinine was detected (≥0.005 ng/mL) and 0 otherwise. Subsequently, multiple logistic regression models were developed using stepwise regression with a 10% significance entry criterion and a 5% significance stay criterion to examine the relationship between detected T1 or T3 cotinine and dwelling type after adjusting for other variables. When the overall *F*-test for dwelling type in the final models was significant, Scheffe’s tests for multiple pair-wise comparisons were used to determine which groups were significantly different from one another.

Multiple stepwise linear regression analyses were performed to examine the relationship between cotinine in cord blood and dwelling type after adjusting for other variables of interest. The response variable, cotinine in cord blood, was treated as a continuous variable and undetected points were replaced by the value LOD/2. The final model was selected with a 10% significance entry criterion and a 5% significance stay criterion. A residual analysis was implemented to verify the statistical assumptions of normality and constant variance. When assumptions were not satisfied for the log-transformed data, the non-parametric method was carried out. When the overall *F*-test for dwelling type in the final models was significant, Scheffe multiple pair-wise comparisons were used to determine which groups were significantly different from one another.

Statistical analyses were performed using the software package SAS (Statistical Analysis System) Enterprise Guide 4.2 and R (R Core Development Team). Unless otherwise indicated, a 5% significance level (*α* = 0.05) was implemented throughout.

## Results

### Descriptive statistics and hypothesis testing

Of the 2001 participants initially recruited, 18 subsequently withdrew and asked that their data and biospecimens be destroyed. At T1, 60.6% of the women reported never smoking, 27.3% were former smokers, 6.1% had quit when they found out they were pregnant, 1.3% occasionally smoked and 4.5% reported current daily smoking (Supplemental Table 1). Similar results were observed at T3. The women were not heavy smokers as only three women reported smoking more than one cigarette per day at T1 and one at T3 (data not shown). Women <25 years of age, with a household income ≤$50,000, low education, or born in Canada were more likely to be daily or occasional smokers during the 1st trimester (Supplemental Table 2). Of those women who had not smoked during the 1st trimester of pregnancy (*n* = 1744), 41.9% reported exposure to SHS (3.0% at home, 3.1% in a vehicle, 7.3% at work and 35.7% in public places) (data not shown). Similar percentages for each location were reported for the 3rd trimester (38.4% overall and 2.3, 2.9, 7.2 and 32.7%, at home, in a vehicle, at work and in public places, respectively).

Approximately 50% of the maternal cotinine samples were below the LODs (Supplemental Table 3). Detection rates for tobacco metabolites in meconium ranged from 22.6% for nicotine to 7.8% for 3-hydroxycotinine. Cotinine was detected in approximately 80% of the cord bloods with no significant differences by infant sex or between singleton and the 14 multiple births. Examining only biospecimens with detected levels, there was moderate correlation (0.74) between T1 and T3 cotinine levels, and between cord cotinine and maternal T1 and T3 levels (0.56 and 0.63, respectively) (data not shown).

As expected, current smokers, who reported no SHS exposure, had substantially higher cotinine levels (GM 12.9 ng/ml) than former smokers (GM 0.007 ng/ml) or women exposed to SHS (GM 0.0108 ng/ml) in the 1st trimester (Table 1). There was a general trend towards increasing cotinine concentration with increasing number of smokers in the household (Supplemental Table 4). Among the 689 women who reported never smoking and no SHS exposure, only 60% of their T1 plasma cotinine levels were <LOD, suggesting that about 40% of the women had some exposure that was not reported (mean 0.0285 ng/ml, range 0.0051–1.9 ng/ml) (data not shown).

**Table 1:** Summary statistics of plasma cotinine in 1st (T1) and 3rd (T3) trimesters by self-reported smoking status in that trimester

	T1 Maternal plasma			T3 Maternal plasma		
	Cotinine (ng/ml)			Cotinine (ng/ml)		
Active only (no second hand smoke)	N	% <LOD	MLE GM	N	% <LOD	MLE GM
Never	689	60.38	NA	627	64.91	NA
Former	294	45.92	0.007	275	54.91	NA
Quit during pregnancy	44	25.00	0.026	45	26.67	0.014
Current	22	9.09	12.91	15	0.00	15.27
Second hand only (no smoking during pregnancy)						
Yes	711	43.18	0.011	574	46.52	0.006
No	983	56.05	NA	902	61.86	NA
Vehicle only						
Yes	20	25.00	0.023	15	33.33	0.016
No	983	56.05	NA	902	61.86	NA
Workplace only						
Yes	54	44.44	0.009	50	46.00	0.006
No	983	56.05	NA	902	61.86	NA
Public place only						
Yes	504	47.62	0.008	400	52.50	NA
No	983	56.05	NA	902	61.86	NA
Exposed to both active and second hand smoke						
Yes (includes women who quit during pregnancy)	167	8.38	2.99	131	10.69	1.278
No	983	56.05	NA	902	61.86	NA

NA: geometric mean was not reported because >50% of results were <LOD

*Note*: 1940 women had T1 cotinine measurements and among these, 1049 reported no SHS exposure. At T3, 1683 women had cotinine measurements and among these, 962 reported no SHS exposure

Cord cotinine concentrations were significantly higher in current smokers and those who quit during the pregnancy than never or former smokers, in women exposed to SHS, and in categories of maternal T1 cotinine used to classify smoking status (Supplemental Table 5). About 85% of the meconium samples with detectable cotinine, nicotine or 3-hydroxycotinine were from women who reported current smoking at T3. In addition, the percentage of meconium samples with detectable cotinine was higher when the non-smoking woman was exposed to SHS in a vehicle. If the woman had a T3 plasma cotinine level >3.0 ng/ml (suggesting active smoking according to Benowitz [12]), then at least one nicotine metabolite was detected in about 97% of the meconium samples (data not shown).

### Sensitivity and specificity of self-reports of tobacco exposure

**Fig. 1:**
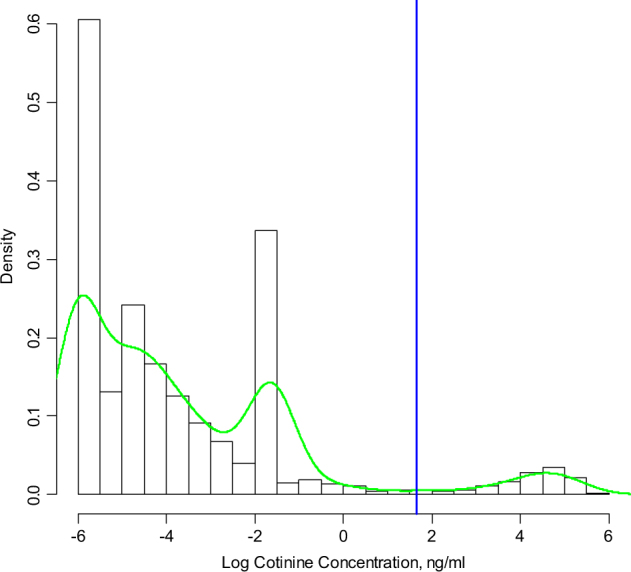
Histogram and kernel density estimate for log cotinine concentration in first trimester, showing cut-point of 5.21 ng/mL and setting values below LOD to a constant LOD/2

In the study population, a trimodal distribution (Fig. 1) was observed in the histogram of T1 log cotinine data when the values below LOD were set to LOD/2. The second peak was caused by imputation of non-detected values (<0.4). The cut-off point for separating active smokers from those not actively smoking (including quitters) was estimated at 5.21 ng/mL (95% CI: 3.35, 8.27). The sensitivity and specificity for self-reported active smoking status at T1 were 85.37 and 99.45%, respectively. Nine out of the ten false negatives were occasional smokers. Similar results were obtained at T3.

We also examined whether the sensitivity differed by maternal characteristics and found that the lowest sensitivity for self-reported active smoking status was found in participants with the highest level of education and income, oldest women and those born outside Canada (Table 2).

**Table 2:** Sensitivity and specificity for self-reported active smoking status using cut-off at 5.21 ng/mL by mothers’ characteristics in first trimester

	Non-active smokers		Active smokers		Sensitivity	Specificity
	<5.21 ng/mL	≥5.21 ng/mL	<5.21 ng/mL	≥5.21 ng/mL		
Characteristic	*n*	*n*	*n*	*n*	%	%
All mothers^a^	1805	18	10	105	85.37	99.45
Maternal age						
<25	102	5	1	30	85.71	99.03
25–29	404	1	5	33	97.06	98.78
30–34	663	5	3	23	82.14	99.55
≥35	636	7	1	19	73.08	99.84
Parity						
0	799	6	6	44	88.00	99.25
1	731	9	2	37	80.43	99.73
>1	273	3	2	24	88.89	99.27
Pre-pregnancy BMI						
<25	1073	11	2	54	83.08	99.81
25–29	369	5	2	18	78.26	99.46
≥30	244	2	4	13	86.67	98.39
Household income ($)						
≤50,000	276	2	6	55	96.49	98.39
50,001–100,000	725	6	2	29	82.86	99.72
>100,000	727	5	2	10	66.67	99.73
Birthplace						
Canada	1458	13	8	98	88.29	99.45
Other	347	5	2	7	58.33	99.43
Education						
High school	119	7	2	44	86.27	98.35
College diploma	503	6	6	47	88.68	98.82
University degree	1181	5	2	14	73.68	99.83

^a^ Among the 1940 women with T1 cotinine data, two of them were missing active smoking information and were not included in this table

### Predictors of plasma cotinine in non-smokers

**Fig. 2:**
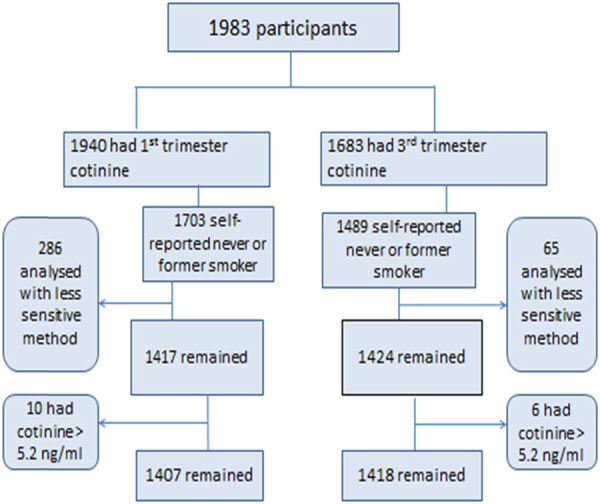
Flow chart identifying non-smoking participants

Women who reported being non-smokers were excluded from this analysis if their cotinine concentration was >5.2 ng/ml (the cut-point for active smokers identified earlier) and the less sensitive method for plasma cotinine was used (Fig. 2). Cord bloods measured with the less sensitive method (*n* = 36) were excluded from the analysis. Cotinine was detected in 58.85% of the remaining T1 samples, 45.77% of the T3 samples and 82.09% of the cord bloods (data not shown).

Table 3 shows that mothers living in an apartment or a townhome have 1.7 times and 1.5 times, respectively, higher odds of detectable cotinine in their T1 maternal plasma than mothers living in a single home after adjusting for other variables. Similar results were obtained for predictors of cotinine at T3 (apartment OR = 1.98; 95% CI 1.39, 2.82), controlling for household income, maternal age and SHS exposure at home, in a vehicle, at work or in a public place (data not shown).

**Table 3:** Multiple logistic regression model for plasma cotinine detected in 1st trimester of non-smokers

Variable	Groups	Multiple logistic regression model (*n* = 1337^a^, H-L *p*-value = 0.87^b^)		Differences^c^
		OR (95% CI)	*P*-value	
Dwelling type	Apartment	1.72 (1.24, 2.39)	0.0005	A
	Townhome	1.52 (1.15, 2.00)		A
	Single Home	Reference		B
Household income			0.0001	
SHS in public places			0.0003	
SHS in vehicle			0.003	
Maternal age			0.027	
SHS in home			0.03	
SHS at work			0.04	

786 plasma samples >0.005 ng/mL cotinine

^a^ 63 missing household income information, 5 missing passive smoking in public information, one participant missing passive smoking in home information and one participant missing both household income and passive smoking in public information, leaving 1337 for analysis in the logistic regression model

^b^ H-L *p*-value: Hosmer-Lemeshow test, *p* > 0.05 indicates a good fit

^c^ Scheffe multiple pair-wise comparison–categories with the same letter are not significantly different from each other

While household income, maternal age, education and exposure to SHS were significantly associated with cord cotinine, no significant difference (*p* = 0.21) was noted among dwelling type groups after adjusting for these other variables (Table 4), even when modelled as a dichotomous variable (detected vs. not). While not statistically significant the direction of the effect for living in an apartment was similar to that observed for maternal blood cotinine (OR = 1.54; 95% CI 0.89–2.66).

**Table 4:** Multiple linear regression model for log cotinine in cord plasma (parametric method)

Multiple regression model (*n* = 1087, *R*^2^ = 0.14)		
Variable	Estimate β	*p*-value
Log(cotinine)		
Intercept	−2.6502	<0.0001
Dwelling type: apartment	0.1538	0.21
Dwelling type: townhome	0.0324	
Dwelling type: single home (reference)		
Household income: <=$50,000	0.3345	0.004
Household income: $50,000-$100,000	0.0800	
Household income: more than $100,000 (reference)		
SHS in public: No	−0.1638	0.01
SHS in public: Yes (reference)		
SHS in vehicle: No	−0.7836	<0.0001
SHS in vehicle: Yes (reference)		
SHS at home: No	−1.3753	<0.0001
SHS at home: Yes (reference)		
Education: High school or less	0.3104	0.01
Education: Some college and college	0.1675	
Education: Undergraduate degree or higher (reference)		
Maternal age: <25	0.2304	0.01
Maternal age: 25–29	0.1593	
Maternal age: 30–34	0.2214	
Maternal age: 35+ (reference)		

## Discussion

In this Canadian cohort of pregnant women, smoking prevalence was low (12% smoked during at least part of the pregnancy) but more prevalent in Canadian-born younger women with less education and income. While approximately 50% of the maternal plasma samples had detectable levels of cotinine, tobacco metabolites in infant meconium were not frequently measured (22.6% > 0.13 ng/g for nicotine; 18.2% > 0.076 ng/g for cotinine; and 7.75% > 0.093 ng/g for 3-hydroxycotinine). In contrast, with similar detection limits (nicotine: 0.946 ng/g, cotinine: 0.070 ng/g, and 3-hydroxycotinine: 0.092 ng/g), an analysis of infant meconium in the Cincinnati HOME Study (*n* = 335) reported the percentages detected were 80.1, 69.6 and 56.4%, respectively, while 61.2% of the women had detectable serum cotinine (>0.015 ng/ml) [6]. The lower detection of tobacco metabolites in MIREC meconium may be due to the substantially higher median maternal cotinine levels in the HOME Study (0.02 ng/ml) compared to MIREC (0.008 ng/ml). However, similar to the HOME Study, elevated tobacco metabolites in meconium (GM 24.01, 23.89, and 36.91 ng/g for cotinine, nicotine and 3-hydroxycotinine, respectively) were observed when T3 maternal plasma cotinine levels exceeded 3.0 ng/ml. As supported by other studies [6, 7, 13], our data suggest that meconium is a useful biological matrix to denote prenatal tobacco exposure in smokers but is less so for those exposed to SHS. As meconium is a complex viscous matrix, incomplete extraction of nicotine and its metabolites bound to chemicals and other compounds in the meconium likely makes it difficult to detect nicotine and cotinine at lower concentrations [14, 15]. Nicotine and cotinine appear to be stable in meconium under multiple freeze-thaw conditions [16]. One of the limitations of measuring concentrations of cotinine in cord blood is that its detection window is hours to days, compared to meconium’s detection window of primarily 3rd trimester [17].

Societal stigma may be responsible for some women being reluctant or uncomfortable reporting their smoking habits, especially during pregnancy [18]. An analysis of US NHANES data using a serum cotinine cut-off of ≥5 ng/mL for non-Hispanic whites found that pregnant women were more likely to not disclose cigarette smoking (22.9%) than non-pregnant women (9.2%) and the rate of non-disclosure varied by demographic characteristics with younger pregnant women 20–24 years of age significantly under-reporting active smoking [19]. We found that older, more educated women were more likely to under-report active smoking, possibly reluctant because they are aware of the hazards of smoking, yet have not been able to quit. In Scotland, under-reporting of active smoking occurred more frequently among pregnant women living in higher socio-economic status (SES) areas (39%) compared with pregnant women in the lower SES regions (22%), with no apparent effect of maternal age [2]. Using a similar cut-point for active smokers (5.3 ng/ml) to ours (5.2 ng/ml), MoBa investigators reported a sensitivity and specificity for self-reported smoking of 82 and 99%, respectively [4]. They also observed the lowest sensitivity in women with the highest education.

There is some evidence to suggest that living in multiunit housing may be associated with a higher risk of being exposed to SHS for pregnant women and their fetuses. Non-smoking women living in apartment buildings were at increased odds (OR 1.72; 95% CI 1.237, 2.392) of having detectable T1 cotinine in their blood, compared to women living in a single home and controlling for exposure to SHS and other socio-economic factors. A similar association, albeit not statistically significant was observed in cord blood. A Rhode Island study of about 190 mother–infant pairs has also reported that non-smoking women living in an apartment were twice as likely (OR 1.95; 95% CI 1.03–3.71) as women in single family homes to have cotinine detected in their serum collected after delivery and three times more likely (OR = 3.17; 95% CI 1.32–5.50) to have detectable cotinine in their cord blood [20]. An analysis of US national survey data also provides evidence that multiunit housing may be a significant source of SHS exposure for children [21], as well as pregnant women and fetuses. One of the limitations of this study is that other potential sources of nicotine such as smokeless tobacco and vaping were not considered in this analysis. It is unlikely that e-cigarettes have contributed to cotinine levels in our population as a 2013 Canadian survey reported that only 3% of females 25–44 years of age used e-cigarettes in the past 30 days [22]. Information was available on whether former smokers or women who quit smoking during the 1st trimester had used any of the following within the past 3 months: a nicotine patch (*n* = 9), nicorettes or other nicotine gum, lozenge or candy (*n* = 5), and medication such as Zyban (*n* = 2). However, we expect that almost all measured cotinine came from tobacco smoking or SHS.

This study is one of the largest prospective studies conducted to date with multiple measures of exposure (self-reports and biomonitoring in multiple matrices) over the course of pregnancy. It adds to the limited body of evidence that non-smoking pregnant women living in multiunit dwellings are at higher risk of being exposed to SHS. As tobacco exposure is a major risk factor for many adverse health outcomes, it is critical to have valid information on active and passive exposure. Since pregnant women and newborn infants represent a particularly susceptible population, evaluating exposures during and after pregnancy using both biomonitoring and self-reported data is informative. Our results suggest that while self-reports are fairly accurate, they may be less so in populations with higher socio-economic status and that living in multiunit housing may be a source of exposure to SHS for non-smoking pregnant women.

## Electronic supplementary material

Supplementary Information
