# Supplementary material for: Exposure to tobacco smoke and validation of smoking status during pregnancy in the MIREC study
Source: J Expo Sci Environ Epidemiol. 2018 Jan 3;28(5):461–9. doi: 10.1038/s41370-017-0011-z (PMC8075994; doi:10.1038/s41370-017-0011-z)
Supplement: Supplementary file 1 — Supplementary Information [file 41370_2017_11_MOESM1_ESM.docx]

**Supplemental Information:**

**Exposure to Tobacco Smoke and Validation of Smoking Status during Pregnancy in the MIREC Study**

**Tye E. Arbuckle, PhD1, Chun Lei Liang, MSc1, Mandy Fisher, MSc1, Nicolas J. Caron, PhD2, William D. Fraser, MD3, 4 and the MIREC Study Group**

Table of Contents

[Results of Reference Materials 2](#_Toc490025481)

[Supplemental Table 1. Self-reported maternal tobacco exposure during 1st trimester of pregnancy according to maternal characteristics. 3](#_Toc490025482)

[Supplemental Table 2. Simple logistic regression results for maternal factors associated with 1st trimester active smoking status (occasional or daily smoker). 4](#_Toc490025483)

[Supplemental Table 3. Descriptive statistics for tobacco metabolites in maternal and cord plasma and meconium. 5](#_Toc490025484)

[Supplemental Table 4. Descriptive statistics of maternal plasma cotinine by active smoking status and number of people smoking in home. Note that “not current smoker” includes women who quit during pregnancy, were former smokers or never smoked. 6](#_Toc490025485)

[Supplemental Table 5. Summary statistics of cotinine in cord plasma and in meconium by self-reported smoking status in the 3^rd^ trimester. 7](#_Toc490025486)

[Supplement Figure 1. : Histogram of the cut-offs from the 10,000 re-samplings. 9](#_Toc490025487)

[Supplemental Figure 2. Histogram of the sensitivities estimated for each cut-off from the 10,000 re-samplings. 10](#_Toc490025488)

[Supplemental Figure 3. Histogram of the specificities estimated for each cut-off from the 10,000 re-samplings. 11](#_Toc490025489)

# Results of Reference Materials

In-house reference materials (IHRM) were prepared using certified standards from a secondary provider independent from those used for calibration (primary). Materials were prepared by spiking blank serum samples with a known concentration of the secondary reference standards. IHRM were frozen and used through the entire study to monitor assay stability. No bias or significant variation was observed during the study. Results shown (average and standard deviation) are from at least 20 measurements and 10 independent runs. Theoretical values were: Plasma cotinine (INSPQ method C-551) low: 10 ng/ml high: 200 ng/ml; Plasma cotinine (INSPQ method C-566) low: 0.1 ng/ml high: 2 ng/ml.

|  |  | 2009 | | 2010 | | 2011 | | 2012 | |
| --- | --- | --- | --- | --- | --- | --- | --- | --- | --- |
|  |  | Average | SD | Average | SD | Average | SD | Average | SD |
| Plasma Cotinine Method C-551 | Cotinine low | 10.38 | 0.68 | 10.50 | 0.89 | 10.47 | 0.83 | 10.71 | 0.49 |
|  | Cotinine high | 209 | 12 | 211 | 14 | 211 | 11 | 203 | 15 |
| Plasma Cotinine Method C-566 | Cotinine low | __ | __ | __ | __ | 0.083 | 0.012 | 0.083 | 0.004 |
|  | Cotinine high | __ | __ | __ | __ | 1.69 | 0.08 | 1.73 | 0.08 |

# Supplemental Table 1. Self-reported maternal tobacco exposure during 1st trimester of pregnancy according to maternal characteristics.

| Characteristics | Active Smoking Status (n=2 missing) | | | | | | Second Hand Smoke Exposure (n=10 missing)^a^ | |
| --- | --- | --- | --- | --- | --- | --- | --- | --- |
|  |  | Never Smoked (n=1202) | Former Smoker (n=542) | Quit During Pregnancy (n=121) | Occasional Smoker (n=27) | Daily Smoker (n=89) |  |  |
|  | N | % | % | % | % | % | N | % |
| **All women** | 1981 | 60.6 | 27.3 | 6.1 | 1.3 | 4.5 | 1734 | 41.9 |
| **Maternal Age** |  |  |  |  |  |  |  |  |
| <25 | 139 | 48.9 | 17.3 | 11.5 | 3.6 | 11.5 | 92 | 70.7 |
| 25-29 | 457 | 59.7 | 23.4 | 8.5 | 2.6 | 5.7 | 379 | 47.8 |
| 30-34 | 709 | 61.8 | 29.5 | 5.1 | 0.9 | 2.8 | 643 | 41.1 |
| ≥35 | 676 | 62.6 | 30.0 | 4.4 | 0.6 | 2.5 | 620 | 35.7 |
| **Parity (n=2 missing)** |  |  |  |  |  |  | (n=2 missing) |  |
| 0 | 873 | 59.9 | 27.0 | 7.3 | 1.7 | 4.0 | 755 | 47.0 |
| 1 | 799 | 63.0 | 27.3 | 4.8 | 0.9 | 4.1 | 716 | 40.4 |
| >1 | 307 | 56.7 | 28.7 | 6.2 | 1.6 | 6.8 | 261 | 32.6 |
| **Pre-pregnancy BMI (n=146 missing)** |  |  |  |  |  |  | (n=116 missing) |  |
| <25 | 1163 | 60.8 | 28.0 | 6.4 | 1.0 | 3.9 | 1027 | 38.8 |
| 25-29 | 404 | 63.4 | 26.5 | 5.0 | 1.0 | 4.2 | 361 | 40.2 |
| ≥30 | 269 | 56.9 | 29.0 | 7.8 | 1.9 | 4.5 | 230 | 55.2 |
| **Household income (n=93 missing)** |  |  |  |  |  |  | (n=76 missing) |  |
| ≤50,000 | 347 | 48.4 | 24.8 | 8.9 | 3.8 | 14.1 | 254 | 59.5 |
| 50,001-100,000 | 785 | 61.3 | 27.5 | 7.3 | 1.0 | 2.9 | 695 | 45.2 |
| >100,000 | 756 | 66.0 | 28.7 | 3.7 | 0.5 | 1.1 | 709 | 33.4 |
| **Birthplace** |  |  |  |  |  |  |  |  |
| Canada | 1610 | 58.4 | 28.3 | 6.7 | 1.3 | 5.3 | 1388 | 45.0 |
| Other | 371 | 70.6 | 23.2 | 3.5 | 1.6 | 1.1 | 346 | 30.9 |
| **Education (n=2 missing)** |  |  |  |  |  |  | (n=1 missing) |  |
| High school or less | 175 | 40.0 | 21.7 | 12.0 | 4.6 | 21.7 | 108 | 54.6 |
| Some college or college | 571 | 51.3 | 29.8 | 9.6 | 1.8 | 7.5 | 460 | 49.4 |
| University degree or higher | 1233 | 68.0 | 27.0 | 3.7 | 0.7 | 0.7 | 1165 | 38.1 |

^a^ excludes women who reported that they quit smoking during pregnancy or were occasional or daily smokers

# Supplemental Table 2. Simple logistic regression results for maternal factors associated with 1st trimester active smoking status (occasional or daily smoker).

| **Variable** | **Groups** | **OR (95% CI)** | **p-value** | **Differences^a^** |
| --- | --- | --- | --- | --- |
| Maternal age | <25 | 8.95 (4.96, 16.15) | <0.0001 | A |
|  | 25-29 | 2.83 (1.64, 4.89) |  | B |
|  | 30-34 | 1.19 (0.66, 2.13) |  | C |
|  | >=35 | reference |  | C |
| Parity |  |  | 0.09 |  |
| Pre-pregnancy BMI |  |  | 0.60 |  |
| Household income ($) | <=50,000 | 13.49 (7.16, 25.40) | <0.0001 | A |
|  | 50,001-100,000 | 2.55 (1.30, 5.00) |  | B |
|  | >100,000 | reference |  | C |
| Maternal place of birth | Canada | 2.54 (1.32, 4.91) | 0.006 | A |
|  | Other | reference |  | B |
| Maternal Education | High school or less | 25.50 (14.21, 45.78) | <0.0001 | A |
|  | Some college or college | 7.32 (4.20, 12.76) |  | B |
|  | University degree or higher | reference |  | C |

^a^ Categories with different letters are significantly different in pair-wise comparisons.

# Supplemental Table 3. Descriptive statistics for tobacco metabolites in maternal and cord plasma and meconium.

| **Smoking metabolite and matrix** | **Sex** | **N** | **% < LOD^a^** | **KM Median^b^**  **(95% CI)** | **MLE GM^c^**  **(95% CI)** | **95th percentile** | **Maximum** |
| --- | --- | --- | --- | --- | --- | --- | --- |
| Cotinine  (1^st^ trimester, ng/ml) |  | 1940 | 46.08 | 0.008  (0.007, 0.009) | 0.008  (0.007, 0.01) | 38 | 290 |
| Cotinine  (3^rd^ trimester, ng/ml) |  | 1683 | 51.22 | NA | NA | 5.9 | 240 |
| Cotinine  (cord, ng/ml) | All | 1384 | 18.57 | 0.012  (0.011, 0.013) | 0.015  (0.013, 0.017) | 3.8 | 110 |
|  | Male | 734 | 18.94 | 0.011  (0.010, 0.012) | 0.015  (0.013, 0.018) | 7.8 | 110 |
|  | Female | 649 | 18.18 | 0.012  (0.011, 0.013) | 0.015  (0.012, 0.017) | 0.74 | 79 |
| Cotinine  (meconium, ng/g) | All | 1525 | 81.84 | NA | NA | 5.5 | 270 |
|  | Male | 803 | 80.57 | NA | NA | 19 | 270 |
|  | Female | 722 | 83.24 | NA | NA | 1.1 | 270 |
| Nicotine  (meconium, ng/g) | All | 1523 | 77.41 | NA | NA | 5.3 | 260 |
|  | Male | 803 | 74.84 | NA | NA | 21 | 250 |
|  | Female | 720 | 80.28 | NA | NA | 2.15 | 260 |
| 3-Hydroxycotinine  (meconium, ng/g) | All | 1523 | 92.25 | NA | NA | 12 | 310 |
|  | Male | 803 | 91.78 | NA | NA | 26 | 310 |
|  | Female | 720 | 92.78 | NA | NA | 1.55 | 290 |

^a^ Percentage less than the limit of detection (LOD). Note % < LOD for cotinine is calculated by combining the frequency of non-detects using the different analytical methods.

^b^  Empirical median using Kaplan-Meier method (censoring method).

^c^ Geometric mean using Maximum Likelihood estimation method (censoring method).

NA: % < LOD too high to calculate valid statistics

# Supplemental Table 4. Descriptive statistics of maternal plasma cotinine by active smoking status and number of people smoking in home. Note that “not current smoker” includes women who quit during pregnancy, were former smokers or never smoked.

| aliquot | smoking status | number of people smoking in home | n | % <LOD | Geometric Mean^a^ (ng/ml) |
| --- | --- | --- | --- | --- | --- |
| 1^st^ trimester | Current smoker | 0 | 58 | 5.2 | 34.0 |
|  |  | 1 | 15 | 0 | 48.5 |
|  |  | 2 | 32 | 0 | 84.6 |
|  |  | 3 | 8 | 0 | 94.2 |
|  |  | 4 | 2 | 0 | 40.6 |
|  | Not-current smoker | 0 | 1760 | 49.8 | NA |
|  |  | 1 | 47 | 19.1 | 0.155 |
|  |  | 2 | 9 | 33.3 | 0.062^b^ |
|  |  | 3 | 3 | 0 | 1.308 |
|  |  | 5 | 1 | 0 | 1.100 |
| 3^rd^ trimester | Current smoker | 0 | 45 | 2.2 | 31.560 |
|  |  | 1 | 9 | 0 | 33.7 |
|  |  | 2 | 18 | 0 | 63.4 |
|  |  | 3 | 5 | 0 | 69.9 |
|  | Not-current smoker | 0 | 1558 | 54.9 | NA |
|  |  | 1 | 33 | 12.1 | 0.132 |
|  |  | 2 | 10 | 0 | 0.092 |

^a^ Calculated Geometric mean using LOD/2 method

^b^ Due to high percentage of non-detects, the result should be interpreted with caution

# Supplemental Table 5. Summary statistics of cotinine in cord plasma and in meconium by self-reported smoking status in the 3^rd^ trimester.

| **Smoking Status** | **Cord** | | | | | **Meconium** | | |
| --- | --- | --- | --- | --- | --- | --- | --- | --- |
|  | **Cotinine (ng/ml)** | | | | | **Cotinine (ng/g)** | | |
| **Active Only (no second hand)** | N | %<LOD | MLE GM | P value | | N | %<LOD | MLE GM |
| Never | 503 | 22.27 | 0.009 | A | <0.0001 | 557 | 92.64 | NA |
| Former | 220 | 21.36 | 0.010 | A |  | 251 | 85.66 | NA |
| Quit during Pregnancy | 33 | 6.06 | 0.030 | B |  | 41 | 68.29 | NA |
| Current | 14 | 0.00 | 13.6 | C |  | 13 | 15.38 | 13.0 |
| **Second Hand Only (excludes women who quit during pregnancy and current smokers)** |  |  |  |  | |  |  |  |
| Yes | 447 | 17.90 | 0.014 | A | <0.0001 | 492 | 83.13 | NA |
| No | 723 | 21.99 | 0.010 | B |  | 808 | 90.47 | NA |
| **Vehicle Only** |  |  |  |  | |  |  |  |
| Yes | 13 | 7.69 | 0.020 | A | 0.006 | 13 | 61.54 | NA |
| No | 723 | 21.99 | 0.010 | B |  | 808 | 90.47 | NA |
| **Workplace Only** |  |  |  |  | |  |  |  |
| Yes | 42 | 11.90 | 0.016 | A | 0.007 | 43 | 88.37 | NA |
| No | 723 | 21.99 | 0.010 | B |  | 808 | 90.47 | NA |
| **Public place Only** |  |  |  |  | |  |  |  |
| Yes | 311 | 20.26 | 0.011 | A | 0.005 | 348 | 88.79 | NA |
| No | 723 | 21.99 | 0.010 | B |  | 808 | 90.47 | NA |
| **(Current or Quit) and SHS** |  |  |  |  |  |  |  |  |
| Yes | 102 | 6.86 | 0.59 | A | <0.0001 | 99 | 27.27 | 1.239 |
| No | 723 | 21.99 | 0.010 | B |  | 808 | 90.47 | NA |
| **Plasma Cotinine (1st trimester)^a^ (ng/ml)** |  |  |  |  | |  |  |  |
| Active (>3.0) | 80 | 1.25 | 11.0 | A | <0.0001 | 86 | 4.65 | 21.0 |
| Not Active (<0.4 old method) | 228 | 36.84 | 0.01 | B |  | 236 | 87.71 | NA |
| SHS (0.005-3.0) | 648 | 11.88 | 0.014 | C |  | 717 | 79.64 | NA |
| Unexposed (<0.005) | 406 | 22.41 | 0.009 | B |  | 458 | 96.07 | NA |
| **Plasma Cotinine (3^rd^ trimester)^a^ (ng/ml)** |  |  |  |  | |  |  |  |
| Active (>3.0) | 74 | 1.35 | 9.2 |  | | 71 | 1.41 | 24.0 |
| Not Active (<0.4 old method) | 54 | 74.07 | NA |  |  | 55 | 83.64 | NA |
| SHS (0.005-3.0) | 582 | 11.17 | 0.015 |  |  | 614 | 78.01 | NA |
| Unexposed (<0.005) | 589 | 23.77 | 0.008 |  |  | 684 | 94.44 | NA |

NA: geometric mean was not reported because % <LOD was larger than 50%

^a^ These are various cut-points based on the literature for “active” (Benowitz et al., 2009; Braun et al., 2010) and our LODs

# Supplement Figure 1. : Histogram of the cut-offs from the 10,000 re-samplings.

# Supplemental Figure 2. Histogram of the sensitivities estimated for each cut-off from the 10,000 re-samplings.

# Supplemental Figure 3. Histogram of the specificities estimated for each cut-off from the 10,000 re-samplings.
